# Salicylic acid and kaolin effects on pomological, physiological, and phytochemical characters of hazelnut (*Corylus avellana*) at warm summer condition

**DOI:** 10.1038/s41598-021-83790-0

**Published:** 2021-02-25

**Authors:** Marziyeh Khavari, Reza Fatahi, Zabihollah Zamani

**Affiliations:** Department of Horticulture Science, Faculty of Agriculture, University of Tehran, Karaj, 31587 Iran

**Keywords:** Light responses, Photosynthesis, Plant breeding, Plant molecular biology, Plant physiology, Plant reproduction, Plant signalling, Plant stress responses

## Abstract

Climate change and population increase are two challenges for crop production in the world. Hazelnut (*Corylus avellana* L.) is considered an important nut regarding its nutritional and economic values. As a fact, the application of supporting materials as foliage sprays on plants will decrease biotic and abiotic stresses. In this study, the effects of salicylic acid (0, 1 mM and 2.5 mM) and kaolin (0, 3% and 6%) sprays were investigated on morphological, physiological, pomological, and biochemical characteristics of hazelnut. The results showed that 1 mM salicylic acid and 6% kaolin had the best effects on nut and kernel weight compared to control. Biochemical parameters such as chlorophyll *a, b, a* + *b*, and carotenoid contents showed that salicylic acid and kaolin improved pigment concentration. Proline and antioxidant contents such as phenolic acids, SOD, APX, and CAT enzyme activities increased by these applications. On the other hand, lipid peroxidation, protein content, and H_2_O_2_ content were decreased. Based on the tolerance index result, Merveille de Bollwiller cultivar showed the highest tolerance while 'Fertile de Coutard' had the lowest value. Therefore, hazelnut performance may be improved through exogenous application of the signaling (salicylic acid) and particle film (Kaolin) compounds in warmer climates.

## Introduction

Nowadays, nuts are more important in the human diet because of their nutritional values^1,2^, and hazelnut are among the most popular tree nuts. The world market of hazelnut witnessed an upward trend, which it reached nearly 1,05 Million tons worldwide in 2017^3^. Because of hazelnut nutritional value, The consumption of hazelnut has been increased in sales^4^. Almost 90% of hazelnut products have been used in the confectionery and chocolate industry, and the remaining are sold in-shell for fresh consumption^5^. Turkey and Italy are leading producer countries, with approximately 60% of the whole global production^3^. Iran produced nearly 18,000 tonnes of hazelnuts in 2017, ranking as 11th country in world production^3^.

Hazelnut (*Corylus avellana* L.) production has been restricted to humid temperate areas with a moderate summer climate. Also, hazelnut trees are sensitive to heat stress in warm summers^6^. As expected, the temperature in the future will be raised approximately 1.5 ºC by 2030, according to prediction obtained from weather modeling. New production management practices are needed in these climatic conditions, and new genotypes to overcome^7,8^. Strategies such as selecting tolerant cultivars, technical management practices, and the use of exogenous protectants for mitigating heat-induced damages are essential to reduce the impacts of high temperatures on horticultural products. Technical management methods include shading and mist irrigation systems, which minimize intense light and high temperatures. However, these techniques need higher technology and abundant water and might spread some fungi diseases^9^.

Currently, particle films are inexpensive and facile ways to decrease the canopy temperature in orchards^10^. Kaolin (KA), an aqueous formulation made from inert clay chemical (Al_4_Si_4_O_10_(OH)_8_)^11^, is formulated for mitigating the solar damage by coating the surface of leaf and fruit. Besides, it improves photosynthesis rate, gas exchange, net CO_2_ assimilation, fruit color, yield, and post-harvest quality reported in olive, walnut, apple, mango, pomegranate, grape, tomato, and berry^12–19^. Kaolin also increases the antioxidant capacity, secondary metabolites such as phenolic content and phenylpropanoids and flavonoids^20–23^.

Salicylic acid plays a crucial role in response to abiotic stresses, and its external application at a suitable concentration can improve the plant’s antioxidant system^24^. SA significantly affects the biosynthesis of supportive compounds such as polyamines, proline, and heat stress proteins^25–28^. Moreover, SA pretreatment revealed the alleviation of oxidative stress as an effective protectant under heat, UV, drought, and salinity stresses^29^. The SA signaling has affected photosystem function by improving the Rubisco activity and net photosynthesis rate (Pn) at heat stress^30–33^. SA treatments were also stimulatory for antioxidant systems like proline, phenolic content, and antioxidant enzyme activities (SOD, APX, CAT) and inhibitory on H_2_O_2_, MDA, and electrolyte leakage (EL) under heat stress in pea, strawberry, olive, grape, cotton, and rapeseed^34–36^.

This study was conducted to understanding the effects of exogenous application of KA, SA alone or in combination on a wide range of pomological and physicochemical properties of hazelnut. This study aimed to protect hazelnut orchards under heat stress conditions, considering the climate change trends in recent years in many parts of the world.

## Results and discussion

### The pomological characters of nuts and kernels

Merveille de Bollwiller cultivar was showed the highest weight of nut and kernel while Segorbe the lowest values in these traits (Table 1). SA and KA application did not affect the pomological characteristics such as sphericity, geometric mean diameter, and nut volume. However, the kernel properties like sphericity of the kernel (%), geometric mean diameter, and the kernel volume were affected compared to control under SA and KA treatments (supplementary data, No.1). The results illustrated in Table 2 and KA treatment at the concentration of 6% increased the mean weight of nut and kernel in all varieties, nearly 5.6% and 11.2%. The previous study on Tonda Giffoni hazelnut reported that 3% KA improved the weight of fresh nut and kernel by nearly 21% and 30%^37^. As some previous studies on mango^12^, apple^14,38^, walnut^18^, olive^39,40^ and tomato^16^, and in red-skinned wine grape^19^ suggested KA showing an increase or improvement in weight yield. These effects might be increased by light reflection and canopy evapotranspiration. However, KA reduced leaf temperature and less water stress. Consequently, increased photosynthesis efficiency reduced oxidative stress and supplied more energy to improve the material transfer to the fruits as sink^14,38,41,42^.

**Table 1:** Means comparison of different cultivar for pomological traits (N- weight (weight of 100 nuts), K-weight (weight of 100 kernels)), RWC (leaf relative water content), EL (electrolyte leakage), pigments contents including chlorophyll a, b, a + b, and carotenoid content of hazelnut leaves during summer. Different letters indicate significant difference between treatments at *p* value ≤ 0.05.

Cultivar	N- weight (g)	K- weight (g)	RWC (%)	EL (%)	*chl* a µg/mg FW	*chl* b µg/mg FW	*Chl a* + *b* µg/mg FW	Carotenoid µg/mg FW
Fertile de Coutard	195.92^c^	93.39^b^	75.627^d^	49.13^a^	3.08^e^	3.05^a^	6.13^ab^	1^cd^
Ronde de Piemant	185.19^d^	90.01^c^	80.84^c^	45.69^b^	3.46^a^	2.72^b^	6.18^a^	1.17^e^
Segorb	160.32^f^	75.97^e^	61.15^e^	45.56^b^	3.43^a^	2.49^c^	5.93^bc^	1.42^c^
Long de Espagne	215.14^b^	84.07^d^	86.18^a^	44.58^bc^	3.18^b^	2.54^c^	5.73^d^	1.55^b^
Negret	195.81^e^	76.58^e^	83.83^b^	43.72^c^	3.56^a^	2.48^c^	6.05^a-c^	1.78^a^
Merveille de Bollwiller	227.25^a^	103.23^a^	85.17^ab^	44.54^bc^	3.07^b^	2.83^b^	5.91^cd^	1.49^bc^

**Table 2:** Means comparison for effects of SA and KA treatments on pomological traits (N- weight (weight of 100 nuts), K-weight (weight of 100 kernels)) RWC, EL, Photosynthesis pigments (chl. a, b, total, carotenoids) of hazelnut leaves during summer. Values are the mean ± SEM. Different letters indicate significant difference between treatments at *p* value ≤ 0.05.

Treatments	N- weight (g)	K- weight (g)	Rwc (%)	El (%)	Chl a µg/mg FW	Chl b µg/mg FW	Chl a + b µg/mg fw	Carotenoid µg/mg FW
Control	186.48^e^	81.17^d^	75.68^d^	49.98^a^	2.84^ef^	^2.26^	5.1^ g^	1.31^d^
KA3%	193.99^b-d^	87.49^c^	76.73^d^	46.04^b-d^	2.71f	^2.69cd^	5.4f	1.11^e^
KA 6%	197.1^a-c^	85.91^a^	83.83^a^	44.23^ef^	3.14^d^	^2.8bc^	5.94^ cd^	1.4^ cd^
SA 1 (mm)	197.77^abs^	86.95^bc^	83.62^a^	44.52^d-f^	3.48^c^	^2.59de^	6.08^c^	1.71^ab^
SA 1(mm) * KA 3%	193.73^b-d^	91.44^bc^	80.54^b^	44.95^c-e^	3.65^c^	^2.71cd^	6.37^b^	1.47^c^
SA 1(mm) * KA 6%	199.71^a^	91.19^ab^	81.45^b^	43.29^f^	3.86^a^	^3.02a^	6.83^a^	1.76^a^
SA 2.5(mm)	189.91^de^	87.62^a^	75.33^d^	46.79^b-d^	2.91^e^	^2.75b-d^	5.66^e^	1.28^d^
SA 2.5(mm) * KA 3%	193.03^ cd^	86.46^c^	78.51^c^	46.32^bc^	3.27^d^	^2.49e^	5.77^de^	1.4^ cd^
SA 2.5(mm) * KA 6%	191.4^d^	86.62^c^	73.51^e^	43.71^ef^	3.80^b^	^2.91ab^	6.78^a^	1.62^b^

SA treatment increased nuts and kernels' weight by approximately 6% and 7.2%, respectively, compared to control. Increasing the dry weight of nuts and kernels under SA treatment may reduce the effects of summer heat stress, especially in July and August, by improving the antioxidant system and enhancing photosynthesis. These results follow the studies on peas and olives under the stress of ultraviolet radiation and high temperature^32,39,43^. The results are also in line with the study on strawberry when 1 mM SA was applied at high temperatures under greenhouse conditions that reduced the oxidative stress of treated plants by 10%^44–46^. Plant response to SA seems to be related to concentration, stress intensity, and plant species; however, herbaceous plants' response may vary with the woody perennial plants. Table 2 shows that 1 mM SA and 6% KA had a meaningful increase in nut and kernel's mean weight by approximately 7.1% and 12.7%, respectively. One of the kaolin drawbacks is removing kaolin from shell consumption, but it is not important for kernel consumption. In general, SA and KA's application on the leaves under these treatments seems to enable more photosynthesis and, consequently, more reserves in nut and kernel dry weights.

### Leaf relative water content

As shown in Table 1, 'Fertile de Coutard' leaves was showed the highest Relative water content (RWC) and ‘Segorb' the lowest. High RWC is an indicator for determining the stress-tolerant cultivars. Lossing water from cells will cause lower turgidity and eliminates cell growth. Tolerant cultivars are attributed to having higher RWC, meaning osmotic regulation by modulating stress in the endoplasmic reticulum^47,48^. The current study showed that KA increased RWC by nearly 12% compared to the control. In research on olives, it has shown that KA at 5% increased RWC by approximately 8% compared to control, depending on the seasonal period of experiment^39,49^. KA is likely to increase the available water due to the reductions in leaf temperature, transpiration, and evaporation of treated hazelnut leaves under summer stress. The same research aim on the hazelnut showed that 4% KA caused 23% improvement in RWC in September compared to control. As represented in Table 2, treatment with 1 mM SA was able to better preserve water content by up to 15% compared with the control^27,30,50,51^. The low range of RWC reported in this study is not similar to other research on hazelnut but, however, it would seem that the low value of RWC content stemmed from the climate condition of the research location is stressful, particularly low humidity and high temperature. All of these caused a low value of RWC compared to an optimum condition.

### Electrolyte leakage

Results showed that 'Fertile de Coutard' recorded the highest percentage of electrolyte leakage (EL), and 'Negret' showed the lowest percentage (Table 1). EL was evaluated as an indicator of cellular membranes' ability to maintain the integrity or recover from imposed stresses in plants^52^. KA treatment at 6% reduced the ion leakage by11.5% compared with the control. In some previous studies, KA treatment significantly decreased cell membrane damage and ion leakage due to reduced oxidative stress and increasing photosynthetic performance^21,22,39,41,49^. This result follows the study on hazelnut trees regarding EL that KA reduced 40–30% compared to the control^53^. Also, SA at concentrations of 1 and 2.5 mM reduced EL by 10% and 6.3%, respectively, compared to control ( \* MERGEFORMAT Table 2). In some previous case reports, SA reduced the effects of salinity stress by increasing the absorption of K^+^, Mg^+^ ions, and accumulation of osmotic regulators; conversely, it decreased Cl − and Na + transport^54,55^. SA has reduced EL in grapes and cucumber, nearly 10% under heat stress^30,56,57^. Besides, in our study, the combination of 1 mM SA and 6% KA decreased EL approximately 15.5% compared to control.

### Chlorophyll content

**Figure 1:**
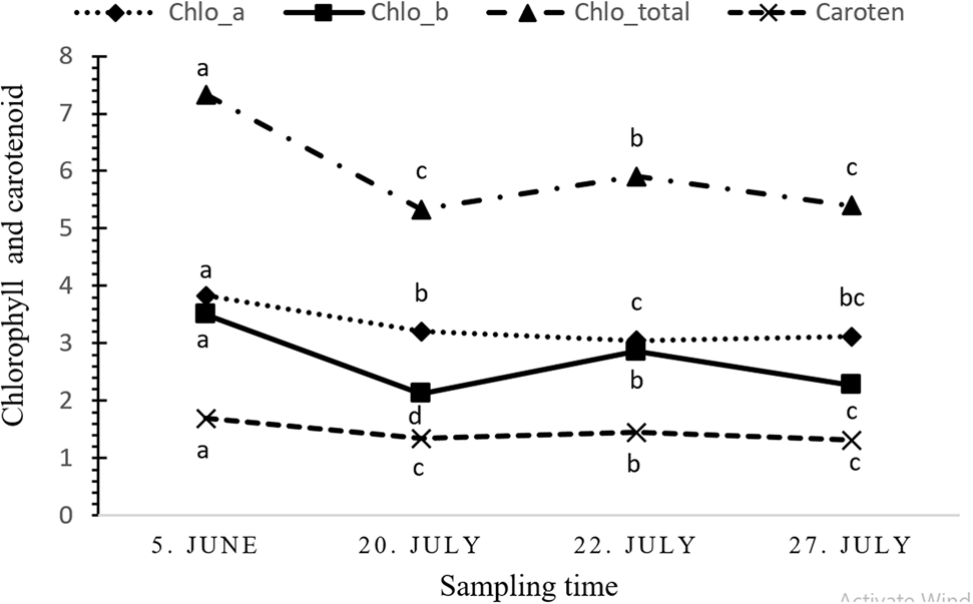
The Means comparison of sampling time for different photosynthetic pigments content (chlorophyll a, b, a + b, and carotenoid) in hazelnut leaves during summer. Each pigment for different times was individually arranged. Different letters indicate a significant difference at a *p* value ≤ 0.05.

The variance analysis for the time of sampling for the effects of SA, KA, and their interaction was significant on the photosynthetic pigments of chlorophyll a (Chl a), chlorophyll b (Chl b), total Chlorophylls (a + b), and carotenoids. The effect of sampling times on the photosynthetic pigments presented in Fig. 1. Results showed a difference in photosynthetic pigments in the first sampling, 1 week after the first foliar application. In the second sampling time, reduced contents of all pigments. In the third stage of sampling, some of the photosynthetic pigments were raised. At the fourth and final sampling time, photosynthetic pigment concentrations were partly decreased. As some previous studies suggested, the decline of chlorophyll content in warm conditions might indirectly be due to obstruction of the activity of the RuBisCO that its failure occurs at temperatures above 35 ℃^58,59^.

As shown in Table 1, Negret cultivar demonstrated the highest amount of *Chl a, a* + *b*, carotenoid; also, 'Fertile de Coutard' showed the highest for chlorophyll b. KA's effect exhibited that 6% KA had an increased effect on photosynthetic pigments such that chl a, chl b, carotenoid, and total chl was 13%, 21%, 17%, and 14% higher, respectively (Table 1). The reflection of intense light caused KA, the reduction of radiation stresses on the leaves by decreasing the leaf temperature (more than 2 ºC) and increased gas exchange, and photosynthesis system activity^42^. These effects might be supported by increased light diffusion, light-reflection, and the index of active photosynthetic radiation, which subsequently enhanced the photosynthesis, chlorophyll content, fraction of vapor pressure, mesophilic leaf conductance of bean, walnut, and almond^60–63^.

As illustrated in \* MERGEFORMAT Table 2, the combination of treatments SA treatment at 1 mM concentration had a considerable positive influence on chl a, chl b, a + b and, carotenoids (33%, 15%, 25%, and 24% increase, respectively). In previous studies for reducing the adverse effects of abiotic stresses, 1 mM or even lower SA concentrations substantially impacted the photosynthesis system^28,29^. Numerous studies have reported that SA improved photosynthesis in grape, pomegranate, bean, and wheat leaves. These treatments might influence the signaling system's activation, increasing the osmotic potential and available water for stomatal conductance, electron transfer to photosynthesis II, and improved the antioxidant system under heat stress conditions^27,30,64–66^. These results support previous research regarding using of SA on tomato (total chlorophyll content increased 10% and Chl a 15%), and rosemary (24% increase in chlorophyll content) at water and salinity stress conditions^67,68^.

Chlorophyll content increased by the interaction of SA and Ka at different concentrations, but 1 mM SA plus 6% Ka resulted in the highest content of Chl a, b, a + b, and carotenoids compared with the control by approximately 45%, 35%, 40%, and 35% respectively (Table 2). So far, the combined effects of SA and KA on photosynthesis has not been reported. It might be possible that the effects of treatments provide the appropriate temperature for photosynthesis by reducing leaf temperature and enhancing the antioxidant activity system.

### Proline, phenols, and MDA content

As presented in Table 3, mean comparisons over sampling times, proline, phenol, and MDA had the lowest amounts at the first sampling (5th June) time. Proline and phenols were increased in the fourth sampling (27th July), about 52% and 7% compred to first sampling (June); while MDA was recorded the highest amount in second sampling (20th July) time.

**Table 3:** Mean comparison of interaction effects for SA and KA treatments for proline (A), phenol (B), and MDA (C) contents in hazelnut leaves during different sampling times in summer. Values are the mean ± SEM. different letters indicate the significant difference between treatments at *p* value ≤ 0.05 (Fisher (LSD)).

Treatment	Proline (µM/g FW)				Phenol (mg/g FW)				MDA (nM/g FW)			
	5 June	20 July	22 July	27 July	5 June	20 July	22 July	27 July	5 June	20 July	22. July	27 July
Control	1.4^p-r^	1.8^k-o^	1.8^p-r^	2.2^e-h^	41.2^m-o^	40.7^no^	40.7^a-e^	40.6^op^	41.9^ab^	47.0^ab^	46.5^a-c^	49.8^a-c^
Ka 3%	1.5^o-r^	1.8^k-n^	1.7^p-r^	2.1^f-j^	41.9^l-o^	43.8^ h-l^	43.2^no^	44.2^d-j^	35.1^a-d^	41.8^c-i^	35.9^f-l^	43.8^f-l^
Ka 6%	1.4^p-r^	1.6^m-p^	1.7^n-r^	1.9^i-l^	41.7^l-o^	44.6^e-j^	44.2^c-h^	44.9^b-g^	33.8^e-k^	29.8^c-g^	30.4^c-h^	39.5^f-m^
SA 1 (mM)	1.9^j-l^	2.0^h-l^	2.4^df^	2.8^bc^	39.1^p^	42.9^no^	41.0^i-l^	44.4^d-i^	37.4^g-m^	44.3^b-e^	37.8^d-j^	37.3^e-j^
SA 1(mM) * Ka 3%	1.8^j-m^	1.8^j-m^	2.3^dg^	3.0^b^	42.3^k-n^	45.0^b-f^	45.2^b-g^	44.9^b-g^	29.5^i-m^	39.6^c-h^	35.4^f-l^	31.2^j-m^
SA 1(mM) * Ka 6%	2.7^bc^	2.5^c-e^	3.5^a^	3.4^a^	43.8^f-k^	45.7^b-g^	45.1^a-d^	46.9^a^	29.8^lm^	43.4^c-f^	28.3^ m^	29.8^j-m^
SA 2.5(mM)	1.2^qr^	1.5^p-r^	2.2^e-i^	2.6^cd^	40.5^op^	44.6^f-k^	43.7^c-h^	44.1^e-j^	24.1^h-m^	44.2^a-c^	52.4^a^	50.5^lm^
SA 2.5(mM) * Ka 3%	1.8^k-n^	2.1^f-k^	2.3^d-f^	3.0^b^	43.6^g-k^	45.5^a-e^	45.5^a-e^	46.2^ab^	43.7^j-m^	40.9^f-l^	38.3^f-m^	36.7^k-m^
SA 2.5(mM) * Ka 6%	2.0^g-k^	1.7^l-q^	1.9^i-l^	2.9^b^	42.7^j-m^	47.0^a-e^	45.5^a^	46.1^abc^	33.8^j-m^	41.6^c-f^	34.3^f-m^	32.1^h-m^
Mean of time	1.7^cd^	1.8^c^	2.1^b^	2.7^a^	41.9^c^	43.8^b^	44.4^a^	44.7^a^	33.81^c^	40.53^a^	36.953^b^	35.58^bc^

The current results were showed KA decreased proline and showed a protective role by smooth the leaf stress away. KA application reduced proline content in walnut and grape by 32% and 38% compared to control^18,22^. Besides, 3% KA treatment were decreased the MDA content in Tonda Giffoni hazelnut nearly 41% compared to control^53^. KA positively impacted the increase of antioxidant compounds such as flavonoids, anthocyanins, and phenolic content in leaf and fruits of grape up to 40% compared with control^17^. KA application was not shown significant changes on the proline in August, whereas it significantly decreased by September^22^. Depending on the plant species, this response may vary even for different locations.

SA significantly affected the biosynthesis activity of enzymes such as Pyrrolline-5-carboxylate reductase and γ-glutamyl kinase under environmental stresses (high salinity and high temperature) and increased protein content^25–27^. Previous studies in other species reported the suppressive effect of SA through manipulation of ion distribution rather than ion accumulation in exposure to salinity^55^. Proline is one of the nitrogen-containing compounds that helps the plants to tolerate abiotic stresses by their involvement in mitigating water uptake and water use efficiency, membrane integrity, enzyme activation, hormonal balance, chlorophyll synthesis, stimulation of photosystems, and CO_2_ assimilation^69^. Several studies believe that proline probably increased as a stress-tolerant osmolyte, while some believe that proline decreased under stress conditions. Proline synthesis and accumulation are considered an indicator of plant damage by temperature and sampling^69–71^. It has also has reported that variation in proline accumulation depends on genotypes and plant species (3–300 fold)^72,73^.

Combined application of 1 mM SA and 6% KA were increased proline(75%) and phenol (11%) Table 3; it also was an efficient treatment for decreasing MDA content in leaves (30%) compared to control. SA could modify the effects of abiotic stresses with the increases in total phenol, antioxidant activity, and proline content in thyme (*Thymus membranaceous*), *Scenedesmus quadricauda*, and cotton^74–76^. In this experiment, the effect of SA on MDA content was exhibited in line with those reported in grapes, cotton, and *Helianthus annus* under heat stress^30,34,35,56,76^*.*

### Antioxidant enzymes activity of leaves

**Figure 2:**
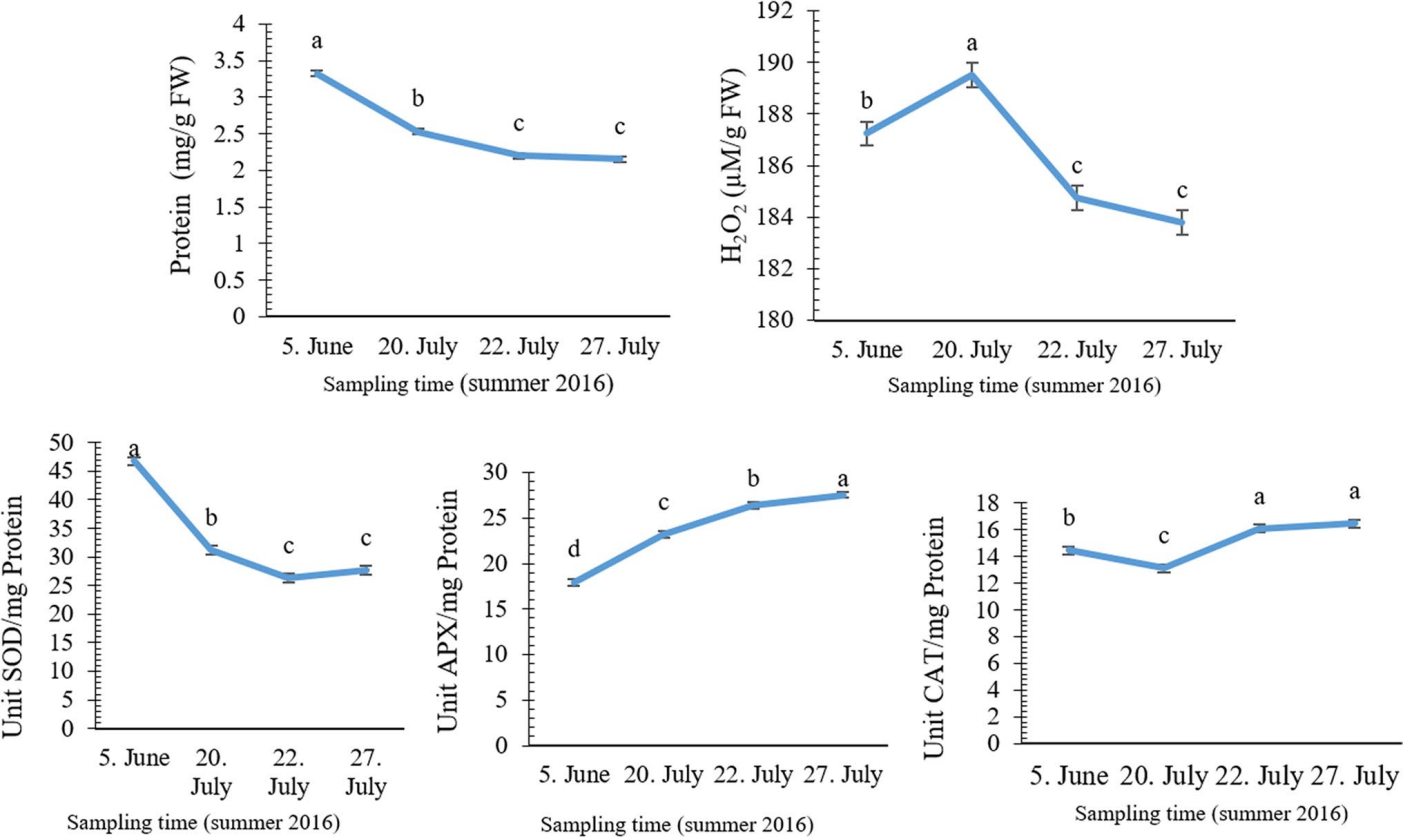
Trends of protein and H_2_O_2_ content and antioxidant enzymes SOD, APX, CAT activity of hazelnut leaves during summer. Different letters indicates significant difference between treatments at *p* value ≤ 0.05 (LSD).

Sampling times showed differences in total protein content, the activity of enzymes SOD, APX, CAT, and hydrogen peroxide content (Fig. 2). The protein content decreased, but H_2_O_2_ increased, possibly destroying total protein by high temperature and oxidative stress in late July. Superoxide dismutase (SOD) activity significantly increased by 55% in July. Also, APX activity showed the lowest value during the first sampling but increased by 40% compared to the first sampling time. The catalase enzyme activity slightly increased in the last two samples. Table 4 shows that the highest total protein content was recorded in Fertile de Coutard and Long de Espagne cultivars. A decline was observed in protein content that seems because of differences in genotypes and metabolites content^47,77,78^. The Segorbe cultivar almost showed the highest activity of SOD, CAT, and APX but the lowest content of H_2_O_2_ and protein during the experiment. Furthermore, the Fertile de Coutard, Long d Espagne, and Merveille de Bollwiller cultivars recorded SOD, APX, and CAT's minimum activity while showing the maximum content of H_2_O_2_ and protein.

**Table 4:** Mean comparison of hazelnut cultivars for biochemical contents, including proline, phenolic acid, MDA, protein, H2O2, and enzymatic activity, including SOD, APX, and CAT in leaves during the experiment. different letters indicate significant difference between treatments at *p* value ≤ 0.05 (Fisher (LSD)).

Cultivar	Proline µM/g FW	Phenolmg/g FW	MDA nM/g FW	Protein mg/g FW	SOD unit SOD/mg protein	APX uUnit APX/mg protein	CAT uUnit SOD/mg protein	H_2_O_2_ µM/g FW
Fertile de Coutard	2.09^c^	45.687^a^	44.81^b^	2.74375^a^	36.91^a^	22.14^c^	13.82^c^	188.52^a^
Ronde de Piemant	1.72^d^	41.250^d^	52.58^a^	2.55^bc^	31.28^b^	23.84^b^	16.54^a^	183.86^c^
Segorb	1.50^e^	42.620^c^	32.69^d^	2.37^d^	27.90^c^	25.80^a^	15.74^b^	185.12^bc^
Long de Espagne	2.27^b^	44.480^b^	36.79^c^	2.62^ab^	35.00^a^	23.03^b^	14.64^bc^	186.92^ab^
Negret	2.714^a^	44.026^b^	29.11^e^	2.57^bc^	31.28^b^	24.06^b^	15.29^b^	185.81^bc^
Merveille de Bollwiller	2.241^bc^	44.112^b^	34.47^cd^	2.46^cd^	35.59^a^	23.68^b^	14.06^c^	187.69^a^

The higher protein content in control treatment may be associated with the stress signaling and transmission, cell membrane instability, heat shock proteins, degradation of cellular proteins, and defense paths^47,79^. Bernardo et al. (2017) reported that KA increased the activity of SOD by 57%, APX by 55%, and CAT by 62% in the plants treated in August, and 5% KA reduced hydrogen peroxide content by 20%. These responses might be related to the stimulating effects of exogenous application of KA on the enzymatic antioxidant defenses^22^. This research showed that SA treatment at 1 mM concentration reduced the total proteins (8%) and hydrogen peroxide production (5%) throughout the experimental period. Also, 1 mM SA treatment increased the activity of SOD, APX, and CAT enzymes by 15%, 20%, and 10%, respectively. The present study's result follows the previous studies performed on other species such as olive, grape, pea, cucumbers, wheat, rosemary, and *Brassica napus*^36,56,57,74^. The application of SA on cotton has also shown the increased activity of SOD and CAT, about 0.49%, and 1.4% under exceptionally high temperature (45/30 °C) compared to optimum temperature (32/20 °C)^35^.

SA reduced oxidative stress and H_2_O_2_ content in many previous reports in plants such as grapes, cotton, and wheat^27,30,76^. Meanwhile, the results of means comparisons for SA and KA's interaction effects on total protein and enzyme activity, including SOD, APX, CAT, and H_2_O_2_ are presented in Table 5. The combined effect of treatments indicated an improvement of antioxidant enzyme activity in 1 mM SA and 6% KA. It also caused the highest reduction in hydrogen peroxide and protein production compared to other treatments and control. Considering the critical role of SA signaling and KA coating to reflect intense light and reduce plant temperature, it might be suggested to apply their combination to reduce stress and stimulate the antioxidant system in high temperatures.

**Table 5:** Means comparison for interaction effects of SA and KA treatments on protein and H_2_O_2_ content and enzymatic activity, including SOD, APX, and CAT of hazelnut leaves during the experiment. Different letters indicate a significant difference at *p* value ≤ 0.05 (LSD).

Treatments	Protein (mg/g FW)	SOD (Unit SOD/mg Protein)	APX (Unit APX/mg Protein)	CAT (Unit CAT/mg Protein)	H_2_O_2_ (µM/g FW)
Control	2.86a	46.77^a^	18.40^e^	9.1212^d^	196.51^a^
Ka 3%	2.58b	33.02^c^	23.95^b^	15.6707^b^	184.96^cd^
Ka 6%	2.16d	27.38^c^	28.54^a^	18.9221^a^	179.94^e^
SA 1 (mM)	2.43bc	28.67^c^	23.81^b^	14.7223^b^	186.76^c^
SA 1(mM) * Ka 3%	2.51b	32.73^b^	23.51^bc^	16.0792^b^	184.49^d^
SA 1(mM) * Ka 6%	2.28cd	25.31^c^	28.99^a^	18.444^a^	180.80^e^
SA 2.5(mM)	2.84a	35.46^b^	20.30^d^	11.5263^c^	192.21^b^
SA 2.5(mM) * Ka 3%	2.54b	33.22^b^	24.19^b^	14.7532^b^	186.51^cd^
SA 2.5(mM) * Ka 6%	2.75a	34.38^b^	22.12^c^	15.917^b^	184.71^cd^

### Heatmap visualization and Pearson correlation analysis among the measured biochemical traits

**Figure 3:**
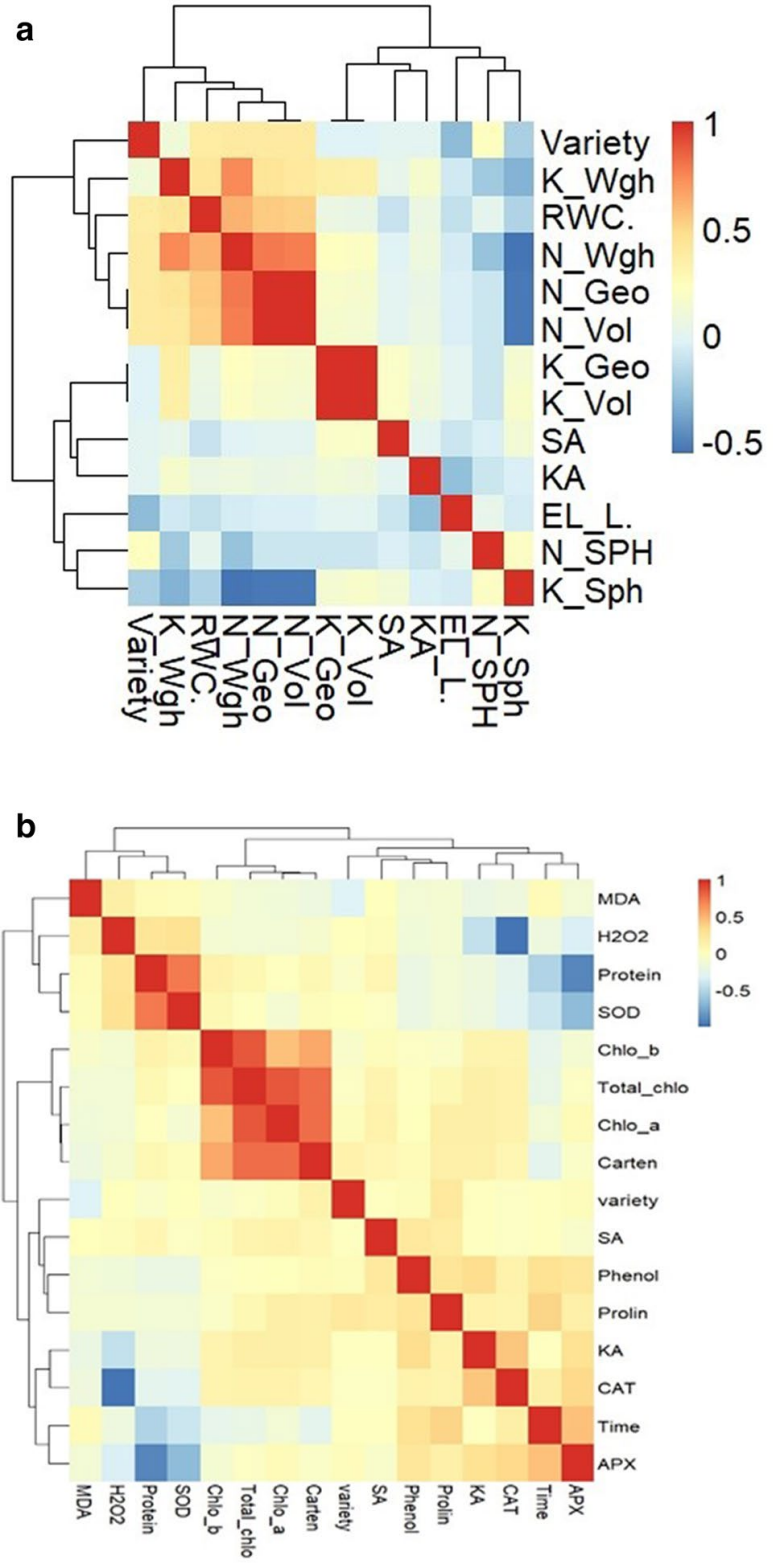
The heatmap visualization and Pearson correlation analysis among the pomological and physiological traits (A) with biochemical traits (B) of hazelnut in the experiment. The relative water content of leaves (RWC), electrolyte leakage of leaves EL_L., mean weight of nut (N_Wgh), the mean weight of kernel (K_Wgh), sphericity of nut (N_SPH), Nut geometry (N_Geo), Nut volume (N_Vol), sphericity of kernel (K_Sph), Geometric of kernel (K_Geo), volume of kernel (K_Vol), Chlorophyll a (Chlo_a), Chlorophyl b (Chlo_b), Chlorophyll content (Total_chlo), carotenoids (Carten), malon de aldehyde (MDA), phenolic content of leaves (Phenol), proline content of leaves (Prolin), protein content of leaves (Protein), superoxide activity of leaves (SOD), ascorbate peroxidase activity (APX), catalase activity (CAT), Hydrogen peroxide (H_2_O_2_).

Heatmaps illustrate the correlation value between hazelnut's physiological, biochemical, and pomological characteristics (supplementary data No.2 and Fig. 3). These relations are essential for selecting valuable traits for breeding trees and selecting tolerant genotypes. Figure 3B shows a significant correlation between chlorophyll content (chlorophyll a, b, and total) and carotenoids, 87–85%. Conversely, there is a slight negative correlation between chlorophyll content, MDA, and H_2_O_2_. Moreover, there is a negative correlation among MDA, protein, and SOD. The relationship between APX, phenol, and CAT is positive but negative for proteins, SOD, and H_2_O_2_. As contrast, As shown in the heatmap results, enzyme activity of SOD, APX, and CAT is correlated with protein, MDA, and H_2_O_2_. Results of the correlation among SOD, APX, and CAT showed these enzymes were affected by sampling time nearly 0.4, 0.45, 0.18. Results showed there is a slight correlation between KA treatments and pomological traits such as the mean weight of kernel and nut. Also, there is a correlation between water content and electrolyte leakage of leaves. The correlations revealed that KA positively regulated the activity of SOD, APX, and CAT by 0.18, 0.29, and 0.43. On the other hand, the KA application negatively correlated with protein content, MDA, and H_2_O_2_ about − 0.17, − 0.21, and − 0.44, respectively. These KA effects were proved that KA alleviated summer stress during the study. La van-hien et al. (2019) reported that SA correlated positively with the expression of signaling genes and subsequently resulted in proline syntheses, like NPR_1_ (nonexpressor of pathogenesis-related) and redox, that resulted in proline synthesis, particularly P5CR genes^36^. The correlation of SA application with phenol content was due to their similar origin and structure^24^. Results show that increased biochemical and enzymatic concentrations have a high relationship with tolerance to environmental stresses such as summer stress (high temperature). Therefore, mechanisms such as antioxidants and enzymatic systems reduce oxidative stress (ROS) in plants and play a critical role in adapting plants to stressed environments^80^. Considering the tolerance of cultivars to environmental stress, it comes due to intrinsic (genetic) and phenotypic ability by regulating physiological and biochemical pathways. Antioxidant enzymes had a positive correlation with total chlorophyll content and a negative correlation with MDA in wheat genotypes^81^. Moreover, the antioxidant defense mechanism plays an essential role tolerance to heat stress in wheat genotypes. It was observed that the activity of SOD, APX, CAT, GR, and POX enzymes were significantly increased in all stages of growth of heat-tolerant cultivars in response to increased stress treatment. In contrast, the sensitive cultivars showed a significant reduction in the activity of CAT^81^.

### The tolerance index

As can be seen in Table 6, Ronde de Piemant and Fertile de Coutard cultivars had the highest tolerance values by considering the biochemical compounds and enzyme activity during the experiment. Nevertheless, Negret showed the highest chlorophyll content. As represented in Table 6, Merveille de Bollwiller recorded the highest value in physiological traits such as RWC and EL and nut and kernel traits, whereas Segorbe showed the lowest. Present results were showed that Merveille de Bollwiller, Long d Espagne, and Negret cultivars had the highest tolerance values in terms of total traits. However, Fertile de Coutard, Ronde de Piemant, and, Segorb cultivars recorded the lowest values.

**Table 6:** The tolerance index calculation of pomological, physiological, and biochemical traits for studied hazelnut cultivars. The relative water content of leaves (RWC), electrolyte leakage of leaves (EL_L)., mean weight of nut (N_Wgh), mean weight of kernel (K_Wgh), chlorophyll a (Chlo_a), chlorophyll b (Chlo_b), chlorophyll content (Total_chlo), carotenoids (Caroten), malon de aldehyde (MDA), phenolic content of leaves (Phenol), proline content of leaves (Prolin), protein content of leaves (Protein), superoxide activity (SOD), ascorbate peroxidase activity (APX), catalase activity (CAT), hydrogen peroxide content (H_2_O_2_).

Traits	Fertile de coutard	Ronde de Piemant	Segorb	Long de Espagne	Negret	Merveille de Bollwiller
RWC%	3.329	3.558	2.692	3.793	3.690	3.749
EL_L%	− 2.162	− 2.011	− 2.005	− 1.962	− 1.925	− 1.960
SUM	1.166	1.547	0.686	1.831	1.765	1.788
N_Wgh	8.618	8.151	7.056	9.469	7.853	10.002
K_Wgh	4.073	3.990	3.316	3.880	3.371	4.544
SUM	12.691	12.141	10.372	13.349	11.223	14.546
Chlo_a	0.005	0.005	0.006	0.006	0.007	0.006
Chlo_b	0.005	0.004	0.004	0.004	0.004	0.005
Total_chlo	0.010	0.010	0.010	0.010	0.011	0.011
Caroten	0.004	0.003	0.004	0.005	0.005	0.005
SUM	0.024	0.023	0.023	0.025	0.028	0.026
MDA	− 1.981	− 2.482	− 1.493	− 1.659	− 1.272	− 1.526
Phenol	2.030	1.828	1.892	1.984	1.966	1.961
Prolin	− 0.095	− 0.079	− 0.070	− 0.105	− 0.122	− 0.104
Protein	0.118	0.106	0.103	0.103	0.105	0.105
SOD	− 1.640	− 1.247	− 1.187	− 1.316	− 1.111	− 1.283
APX	1.027	1.106	1.170	1.102	1.148	1.104
CAT	0.609	0.722	0.715	0.678	0.677	0.603
H_2_O_2_	− 8.299	− 8.103	− 8.111	− 8.172	− 8.171	− 8.287
SUM	− 8.232	− 8.149	− 6.980	− 7.386	− 6.780	− 7.427
Total traits	5.650	5.562	4.102	7.819	6.236	8.934

## Conclusion

The knowledge emerging from this study may be useful to reveal hazelnut cultivars for ameliorating the materials in high temperatures. The laboratory assay effects of salicylic acid and kaolin surveyed at different species and levels that were evaluated how and how much these effects have happened. The study was done in a single year, and research on yield and physiological traits should be replicated.

This study showed that using 6% KA, or 1 mM SA, or even better, their combination can significantly improve hazelnut production. These treatments showed positive effects on leaf physiological properties such as increasing RWC and decreasing electrolyte leakage in field conditions. In this research, 1 mM SA concentration showed the better to improve supportive effect under summer stress. These treatments, such as KA 6% and SA 1 mM alone and their combination improved the photosynthetic system. Interestingly, treatments showed efficient effects on the antioxidant system by reducing H_2_O_2_, proteins, MDA production, and the oppositely increased total phenol, proline, and enzyme activity (SOD, APX, and CAT). Merveille de Bollwiller, Long d Espagne, and Negret cultivars had the highest tolerance values, while Fertile de Coutard, Ronde de Piemant, and Segorb recorded the lowest values.

Results suggest that KA and SA could be useful as inexpensive supplemental materials for reducing harmful effects of new challenges like high temperature during summer in hazelnut orchards.

## Materials and methods

### The field condition

The experiment was conducted at the Horticulture Department's research station, University of Tehran, Karaj, Iran. Karaj is situated about 30 km west of Tehran and the station has an elevation of 1312 m, 35º 49′ 44′' Latitude, and 51º 00′ 21′' Longitude. Six commercial hazelnut cultivars (Merveille de Bollwiller, Long de Espagne, Negret, Segorb, Ronde de Piemant, and Fertile de Coutard) with origins from Spain and Italy were used in this investigation (supplementary data No.3). The soil properties shown in Table 7 and drip‐irrigation system assured fertilization and irrigation. Also, Field management, including pest and weed control, were performed according to the local farming practices. Three uniform 20-year-old trees for each cultivar were selected as replicates (3 × 3 m^2^), and were considered for each treatment. The experiment has been structured as factorial in a Randomized Complete Block Design (RCBD) with three replicates during 2016 summer.

**Table 7:** Physical, chemical, and hydrological characteristics of the soil in this study's experimental site.

Soil parameters (Particle–size analysis)		Soil parameters	
Total sand (2 > ∅ > 0.02 mm)	63%	pH	7.75
Silt (0.02 > ∅ > 0.002 mm)	18%	Electrical conductivity	2.57 dS m − ^1^
Clay (∅ < 0.002 mm)	19%	Field capacity (by weight)	20.6%
Organic matter	0.89%	Wilting point (by weight)	10.2%

For SA treatments, we solved the required SA (Merck) for each concentration in the proper methanol on the stirrer in the first step. Secondly, we solved this solution in some water, but SA solvent did not solve it completely. Then, the water was heated up to nearly 70 ℃ until SA was completely solved and added tween 80 surfactants (0.01%) to decrease water's surface tension for the increase of SA absorption in the leaf surface. Finally, the solution was solved with the water tank for spraying on the hazelnut trees. KA treatment is prepared by a commercial product from Kimia Sabzavar (Al_2_Si_2_O_5_(OH)_4_), which is a white mineral (nearly 2 nm). Treatments included sprays of SA at three levels 0 (control), 1 mM and 2.5 mM and KA at 0 (control), 3%, and 6% with tractor-mounted sprayers. KA and SA applied two times: first sprayed on 5th June at the season of temperature increases, and the second carried out by 20th July at the highest temperature in Karaj.

The study was done in a single year. This experiment's primary purpose was a comprehensive investigation of exogenous protectants on hazelnut trees at different aspects such as production traits, biochemical compounds, etc.

### Meteorological data

All meteorological data have taken from the Alborz Meteorological Synoptic Station located at 500 m distance from the research location. The meteorological data during this experiment and 6 years before the investigation are represented in \* MERGEFORMAT Table 8. The year 2016 has been one of the high-temperature years with 16.18 ºC average temperature of the year. The average relative humidity of the 2016 year showed the lowest value between the years 2010–2016. Conversely, mean sunshine hours per day at this year recorded the highest level. The total precipitation of 2016 was 199.30 mm, one of the low rainfalls during the study. Therefore, considerations of meteorological data of the region during the years can help diagnose climate change and the effects of these parameters on hazelnut production. As shown in Table 9, the mean temperatures in June, July, and August were high during the developing and filling of hazelnut (being 25.12 ºC, 27.39 ºC, and 26.74 ºC, respectively). Some meteorological variables like sunshine hours, pan evaporation, soil temperature, and radiation intensity recorded the highest values during these months. The relative humidity means went down to 28–34% in June, July, and August compared to other months.

**Table 8:** Yearly data of meteorological variables during 2010–2016 obtained from the meteorological institute in Karaj. Maximum temperature (Max.Temp, (°C)), minimum temperature (Min.Temp (°C)), mean temperature (Mean_Temp (°C)), relative humidity (RH_Mean (%)), Sun_shine (Hrs), Evaporation (Evap (Pan mean (mm))), Rainfall (mm), temperature of soil at 50 cm dept (°C) (Temp_soil_50), temperature of soil at 100 cm dept (°C) (Temp_soil_100), Solar radiation (Rad. T.S.R. (kj/m^2^)).

Year	Max.Temp (°C)	Min.Temp (°C)	Mean_Temp (C°)	RH_Mean (%)	Sun_shine (Hrs)	Evap (mm)	Rainfall (mm)	Soil. Temp _50	Soil. Temp _100	Rad. T.S.R
2010	23.65	10.79	17.2	43.80	8.41	7.87	233.50	18.12	18.09	3769.98
2011	21.12	8.82	14.97	49.38	7.93	9.48	446.60	17.41	17.18	2096.21
2012	21.43	9.40	15.42	49.04	8.32	7.97	312.41	17.43	17.08	1898.41
2013	22.41	9.45	15.93	46.27	8.33	8.64	158.70	18.36	17.93	2103.80
2014	22.39	9.75	16.11	44.48	8.14	7.24	188.90	17.86	17.92	13,461.72
2015	22.73	10.10	16.41	44.59	8.14	7.00	215.90	18.53	18.59	37,466.25
mean	22.29	9.72	15.77	46.26	8.21	8.04	259.34	17.95	17.80	10,132.73
2016	22.70	9.69	16.18	42.19	8.57	5.66	199.30	18.64	18.70	18,840.63

**Table 9:** Monthy data of meteorological variables during 2016 obtained by the meteorological institute in Karaj. Maximum temperature (Max.Temp, (°C)), minimum temperature (Min.Temp (°C)), mean temperature (Mean_Temp (°C)), relative humidity (RH_Mean (%)), Sun_shine (Hrs), Evaporation (Evap(Pan mean)), precipation (Precip **(**mm), temperature of soil in 50 cm (°C) (Temp_soil_50), temperature of soil in 100 cm (°C) (Temp_soil_100), Solar radiation (Rad. T.S.R. (kj/m^2^)).

Months	Temp_Max	Temp_Min	Temp_Mean	RH_Max	RH_Min	RH_Mean	Sun Shine (Hrs)	Evap (mm)	Precip	Soil. temp_50	Soil. temp _100	Rad. T.S.R
January	10.11	1.06	5.59	72.7	37.5	52.6	5.9	0.0	19.6	6.5	9.8	9693.8
February	13.18	2.92	8.05	69.6	31.4	47.0	7.1	0.0	12.0	8.0	9.7	13,682.0
March	16.83	5.95	11.39	70	30	46.9	7.6	131.1	37.9	12.5	12.6	17,632
April	21.35	8.50	14.93	77	31	49.6	8.5	136.6	62.4	15.4	14.3	21,113
May	28.78	13.63	21.20	66	21	39.0	9.4	261.0	13.2	22.0	19.5	25,436
June	33.29	16.95	25.12	55	14	28.1	11.0	347.0	0.0	27.2	23.9	27,803
July	35.57	19.21	27.39	63	18	34.3	11.2	347.0	0.0	30.1	26.7	27,228
August	34.23	19.24	26.74	52	15	28.3	11.6	296.4	0.0	30.7	28.0	25,764
September	31.18	15.18	22.99	65	18	36.5	10.3	262.6	0.0	27.7	26.9	22,205
October	23.41	10.13	16.77	66	23	40.7	8.3	151.4	2.4	21.2	22.7	15,935
November	14.89	3.15	9.02	67	28	44.3	6.9	74.5	1.6	14.2	17.9	11,449
December	9.29	0.10	4.70	81.42	41.61	58.87	4.96	0.00	50.00	7.73	12.18	7880.3

### Morphological and pomological measurements for nuts

Nuts were harvested randomly from various parts of trees at the standard ripening time of six genotypes and then dried for 2 weeks. One hundred nuts were used for the evaluation of each genotype. Also, qualitative characteristics were considered based on hazelnut descriptors^82^. The length (L), width (W), and thickness (T) of nuts and kernels were measured. Geometric diameter (D) and sphericity (Ø), the volume of nuts and kernels were calculated using protocols of Mohsenin (1980)^83^.

$${\text{D}} = \left( {{\text{LWT}}} \right)^{{1{/}3}} \quad \O \, = \frac{D}{L} \times 100\quad v = \left( {\frac{{\left( {\pi {\text{LWT}}} \right)}}{8}} \right)$$

$$Kernel\;percentage = \left( {\frac{ kernel\;mass}{{nut\;mass}}} \right) \times 100$$

### The relative water content of leaves

RWC was measured according to Barrs and Weatherley^84^. Leaf laminas sampled with sampling and then weighed (fresh weight (FW)), then placed immediately between two layers of filter paper and immersed in distilled water in a Petri dish for 24 h in a dark place. Turgid weight (TW) was measured after gently removing excess water with a paper towel. Dry weight (DW) was measured after 48 h in the oven drying at 80ºC. Finally, relative turgidity was determined using the following formula:

$${\text{RWC }}\left( {\text{\% }} \right) = \frac{{\left( {{\text{FW}} - {\text{DW}}} \right)}}{{\left( {{\text{TW}} - {\text{DW}}} \right)}} \times 100$$

### Electrolyte leakage of leaves

One month after the second treatment, ten leaves were taken randomly from each plant, thoroughly washed with distilled water, and placed in 50 ml falcons and 20 ml of distilled water added. Then, the flasks were placed on the shaker for 24 h at room temperature, and their electrical conductivity (EC_1_) was measured using an EC meter. Samples were autoclaved for 10 min, and after cooling to room temperature, the electrical conductivity (EC_2_) was measured. Electrolyte leakage is calculated according to the following equation as Membrane Damage^80^.

$${\text{EL\% }} = \frac{{{\text{EC}}1}}{{{\text{EC}}2}} \times 100$$

### Determination of leaf photosynthetic pigments contents

Chlorophylls a, b, a + b, and carotenoid were extracted according to Arnoff^85^ with some modifications. By homogenizing leaf samples (0.05 g) with 2 mL of acetone (80% v/v) followed by centrifuging at 12,000 × *g* for 10 min. Absorbance was measured with a UV–Vis plate-reader at 663 and 645 nm for Chl a and Chl b content, respectively. Carotenoid content was also measured spectrophotometrically using the same plant extract at 470 nm. The Equations for chlorophylls and carotenoids concentrations were calculated according to Lichtenthaler & Porra^86,87^ in µg/mg fresh weight.

### Determination of leaf proline content

Proline (Pro) content was determined, according to Bates et al.^88^. Leaf samples (0.5 g) were homogenized in 5 mL 3% sulfosalicylic acid, and the homogenate was centrifuged at 11.500 × *g* for 12 min. The supernatant (1 mL) was mixed with 1 mL glacial acetic acid and 1 mL acid ninhydrin. After 1-h incubation at 100 °C, the mixture cooled. The developed color was extracted with 2 mL toluene, and the optical density of the chromophore was measured by plate-reader at 520 nm.

### Determination of leaf total phenols

According to Robles-Sánchez et al. (2009) was used to determine the total phenol concentration of hazelnut leaves using the Folin-Ciocalteu reagent.

### Lipid peroxidation of the leaf (as MDA content)

Cell membrane lipid peroxidation/TBARS showing the presence of malondialdehyde was determined based on the relative concentration of MDA content with some modifications^89,90^. Leaf samples (0.5 g) were homogenized in 1.5 ml of 5% (w/v) trichloroacetic acid (TCA), and the homogenate was centrifuged at 12,000* g* for 20 min. A one-third aliquot of the supernatant was mixed with 1 ml of 20% (w/v) TCA containing 0.1% (w/v) TBA, and the mixture put on the boiling water bath for 30 min in 95 ºC, then quickly cooled on ice and centrifuged at 11,000* g* for 10 min. The absorbance of the supernatant was determined at 532 and 600 nm by plate reader.

### Determination of leaf antioxidant enzyme activity and protein

Frozen leaf samples (100 mg) were grounded in liquid N_2_ and extracted in 100 mM phosphate buffer (K_2_HPO_4_ and KH_2_PO_4_,pH = 7) containing 10 mM EDTA and 2% polyvinylpyrrolidone (PVP) in an ice-water bath. The homogenate was centrifuged at 12,000 g for 15 min at 4 ºC, then the supernatant was used for antioxidant enzyme activity assays.

- The measurement of proteins was performed according to the method of Bradford^91^.
- The activity of SOD was determined according to Beauchamp and Fridovich^92^.
- The activity of APX was measured according to the method of Mittler and Zilinskas^93^ with some modifications.
- The activity of catalase measurement and determination of hydrogen peroxide content was according to the Sinha method (1972), by a colorimetric evaluation based on the reaction rate of the enzyme in the potassium dichromate.

### Statistical analyses

1. *Analysis of Variance (ANOVA)*
  Analysis of variance carried out for morphological variables using Minitab software (version 18). Means comparisons were according to LSD (Fisher test) at a confidence level of 95% (*p* value 0.05) by Minitab (version18).
2. *Heatmap*
  The simple correlation coefficients were calculated to determine the relationships between the studied morphological variables using the pearson correlation coefficient with the two-tailed way by RV. 3.2.6 (heatmap package).
3. *The calculation of the tolerant index*
  Firstly, the average for repeats of all traits of every cultivar (genotype) is calculated. Secondly, to eliminate the scale, all of the features are divided into the tenth of each data's maximum value. Finally, each trait's positive or negative effects indicated the summarized value of all traits to a tolerant cultivar's total value.

## Supplementary Information

Supplementary Information 1.

Supplementary Information 2.

Supplementary Information 3.

Supplementary Information 4.
