# Supplementary figures and images for: Salicylic acid and kaolin effects on pomological, physiological, and phytochemical characters of hazelnut (Corylus avellana) at warm summer condition
Source: Sci Rep. 2021 Feb 25;11:4568. doi: 10.1038/s41598-021-83790-0 (PMC7907359; doi:10.1038/s41598-021-83790-0)

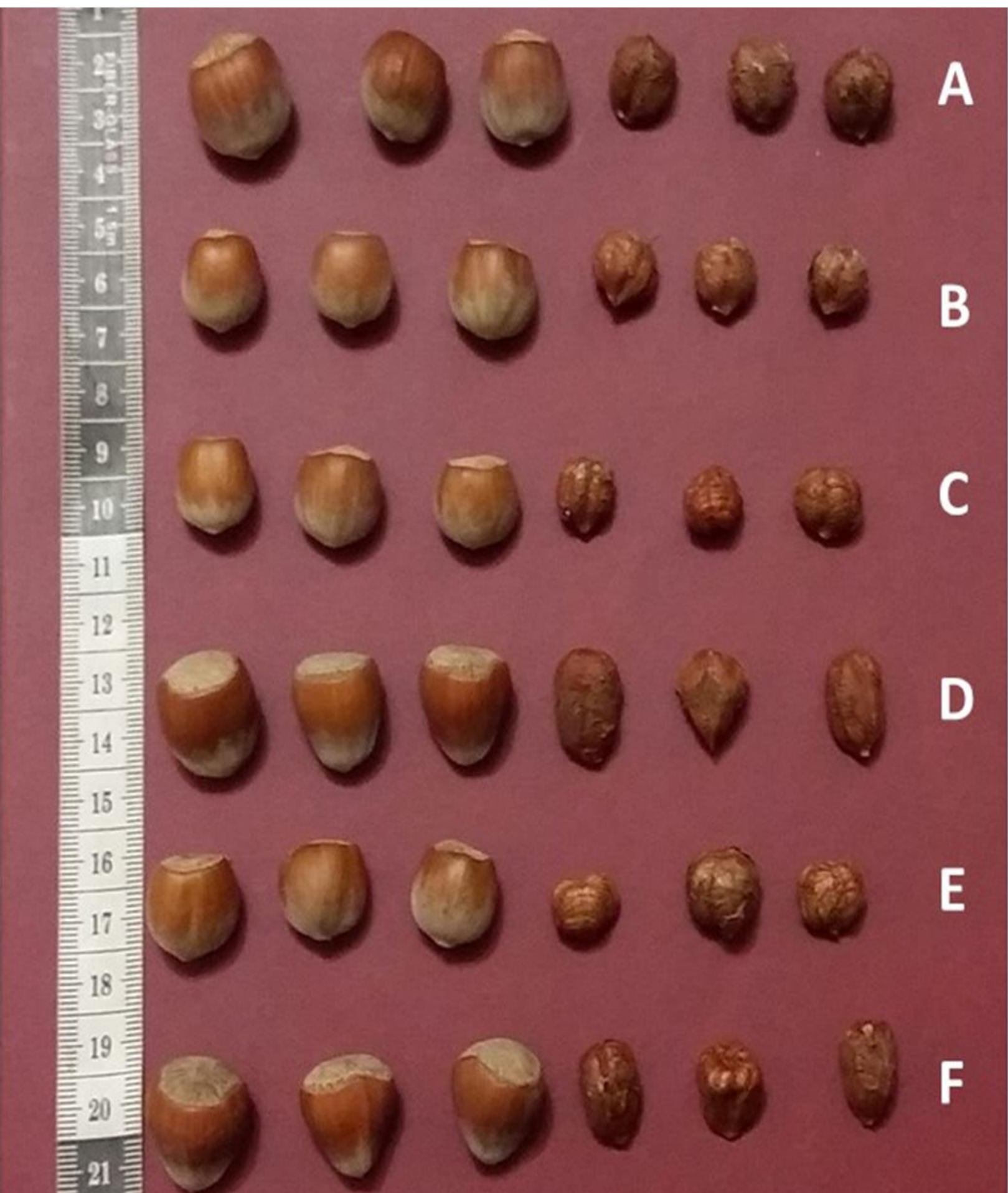

Supplement: Supplementary file 4 — Supplementary Information 4. [file 41598_2021_83790_MOESM4_ESM.pdf]
